# Monocyte Gene Expression Signature of Patients with Early Onset Coronary Artery Disease

**DOI:** 10.1371/journal.pone.0032166

**Published:** 2012-02-21

**Authors:** Suthesh Sivapalaratnam, Hanneke Basart, Nicholas A. Watkins, Stepanie Maiwald, Augusto Rendon, Unni Krishnan, Brigitte M. Sondermeijer, Esther E. Creemers, Sara J. Pinto-Sietsma, Kees Hovingh, Willem H. Ouwehand, John J. P. Kastelein, Alison H. Goodall, Mieke D. Trip

**Affiliations:** 1 Department of Vascular Medicine, Academic Medical Center, Amsterdam, The Netherlands; 2 Department of Hematology, University of Cambridge, Cambridge, United Kingdom; 3 National Health Service Blood and Transplant, Cambridge, United Kingdom; 4 Department of Human Genetics, Wellcome Trust Sanger Institute, Hinxton, United Kingdom; 5 MRC-Biostatistics Unit, Cambridge, United Kingdom; 6 Department of Cardiovascular Sciences, University of Leicester, Leicester, United Kingdom; 7 Leicester NIHR Biomedical Research Unit in Cardiovascular Disease, Glenfield Hospital, Leicester, United Kingdom; 8 Department of Cardiology, Academic Medical Center, Amsterdam, The Netherlands; 9 Heart Failure Research Center, Academic Medical Center, Amsterdam, The Netherlands; University of Tor Vergata, Italy

## Abstract

The burden of cardiovascular disease (CVD) cannot be fully addressed by therapy targeting known pathophysiological pathways. Even with stringent control of all risk factors CVD events are only diminished by half. A number of additional pathways probably play a role in the development of CVD and might serve as novel therapeutic targets. Genome wide expression studies represent a powerful tool to identify such novel pathways. We compared the expression profiles in monocytes from twenty two young male patients with premature familial CAD with those from controls matched for age, sex and smoking status, without a family history of CVD. Since all patients were on statins and aspirin treatment, potentially affecting the expression of genes in monocytes, twelve controls were subsequently treated with simvastatin and aspirin for 6 and 2 weeks, respectively.

By whole genome expression arrays six genes were identified to have differential expression in the monocytes of patients versus controls; *ABCA1*, *ABCG1* and *RGS1* were downregulated in patients, whereas *ADRB2*, *FOLR3* and *GSTM1* were upregulated. Differential expression of all genes, apart from *GSTM1*, was confirmed by qPCR. Aspirin and statins altered gene expression of *ABCG1* and *ADBR2*. All finding were validated in a second group of twenty four patients and controls. Differential expression of *ABCA1*, *RSG1* and *ADBR2* was replicated. In conclusion, we identified these 3 genes to be expressed differently in CAD cases which might play a role in the pathogenesis of atherosclerotic vascular disease.

## Introduction

Cardiovascular disease (CVD) remains the major worldwide cause of morbidity and mortality. Atherosclerosis, the major underlying disease process of CVD, is highly prevalent in western society and its progression is dependent upon genetic and environmental risk factors [1]. Despite currently available therapy targeting known factors, reduction of CVD event rates has never surpassed 50% [2]. A better understanding of the complex pathophysiology of CVD is required for the identification of new therapy targets. Through recent efforts to unravel the molecular basis of coronary artery disease (CAD) by Genome Wide Association Studies (GWAS) and follow up of some of these loci, clues for novel pathways have indeed been discovered, such as the *SORT1* pathway resulting in CAD via lipid metabolism [3].

Another powerful tool for the identification of such novel pathways in disease is Whole Genome Expression profiling. Numerous studies have applied gene expression profiling of carotid, coronary and thoracic arterial wall tissue to identify novel genes involved in atherogenesis [4]. However, only a few studies have determined gene expression profiles in circulating cells. Monocytes are an attractive cell type for this type of approach since they; play a pivotal role in a number of crucial steps in atherogenesis, are in contact with the diseased endovascular lumen and as such may serve as reporter cells, transcribe almost 10,000 genes [5] and are easily accessible both for research and possibly for future diagnostic applications [6].

We performed a study in which we compared monocyte gene expression profiles between young CAD patients with a hereditary background for CVD and controls matched for age, gender and smoking status without a family history. To control for the potential effect of aspirin and statin on differential gene expression, the controls were given these medication to test theeffect on the candidate genes identified from expression profiling.

## Results

### Clinical characteristics

Baseline characteristics of the 22 patients and controls are presented in Table 1. Patients used statin (95%), aspirin (86%), beta-blockers (73%), ACE-inhibitors (41%) and nitrates (23%). None of the controls used any medication. In the 12 healthy subjects given statin and aspirin, LDL-cholesterol levels fell significantly from 2,9±0,6 mmol/L to 1,6±0,6 mmol/L following the 6 week treatment period (p = 0.0002).

**Table 1:** Baseline characteristics Patients and Controls.

	Patients	Controls
	(n = 22)	(n = 22)
Baseline characteristics		
Age ± sd (years)	43,4±3,8	43,1±3,3
Age of Myocardial Infarction ± sd (years)	36,3±5,7	na
Family History of early-onset CVD (n,%)	22 (100)	0
Hx of Hyperlipidemia (n,%)	6 (27)	0
Hx of Hypertension (n,%)	4 (18)	0
Hx of Diabetes (n,%)	0	0
Hx of Smoking (n,%)	13 (59)	7 (32)
Current Smoking (n,%)	6 (27)	6 (27)
Biometrics		
Systolic Bloodpressure ± sd (mmHg)	130,6±19,3	131,6±10,1
Diastolic Bloodpressure ± sd (mmHg)	85,8±10,1	84±10,1
BMI ± sd (kg/m2)	27,6±3,2	25,3±3,1
Lipid and Glucose values		
Total Cholesterol ± sd (mmol/L)	4,3±0,7	5,1±0,9
LDL-cholesterol ± sd (mmol/L)	2,3±0,8	3,1±0,7
HDL-cholesterol ± sd (mmol/L)	1,2±0,3	1,3±0,3
Triglycerides* (mmol/L)	1,3 (0,9–1,6)	1,0 (0,7–1,9)
Apolipoprotein-A ± sd (g/L)	1,4±0,2	1,4±0,2
Apolipoprotein-B ± sd (g/L)	0,9±0,2	0,9±0,2
Glucose ± sd (mmol/L)	5,3±0,6	4,9±0,4
Data are presented as mean ± standard deviation (sd) or median (interquartile range)*.		
Hx indicates history; LDL indicates low-density lipoprotein and HDL indicates high-density lipoprotein.		

In comparison to the study group in the replication group the mean age of first event of CAD and the mean systolic blood pressure was higher. The number of cases who suffered from a MI (54%) and the number of individuals with a history of dyslipidemia was lower. The medication use in the patients was similar to the discovery cohort. Details of the replication group can be found in the supplement (Table S1).

### Differential gene expression and correction for medication use

**Figure 1:**
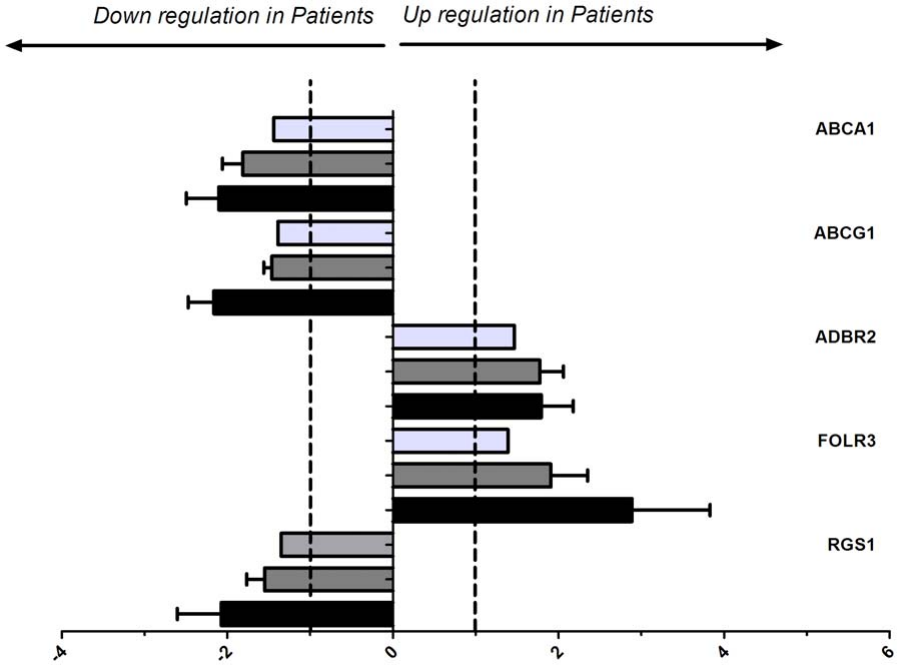
Differential gene expression in patients versus controls validated by qPCR in the discovery set. The X-axis depicts the relative fold changes in gene expression in the patients compared to controls. For each gene the first (pale grey) bar depicts the fold change in microarray expression levels, the second (mid grey) bar shows the change in microarray intensities and the final (black) bar shows the qPCR 2exp ΔΔCT change. Values are given as mean + SD. A relative fold change of ≤1 or −1 indicates no effect.

**Figure 2:**
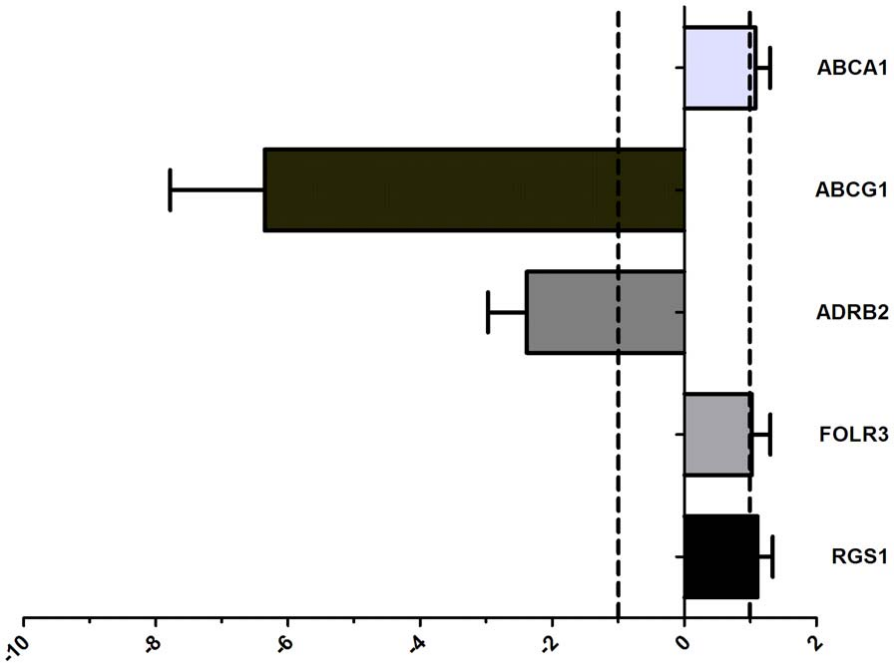
Differential gene expression in controls due to medication. The x-axis the average change in expression following the use of statins and aspirin depicted as 2expΔΔCT. Values are given as mean + SD. A relative fold change of ≤1 or −1 indicates no effect. For ADRB2 and ABCG1 the changes were significant; p = 0.04 and p<0.001 respectively.

**Figure 3:**
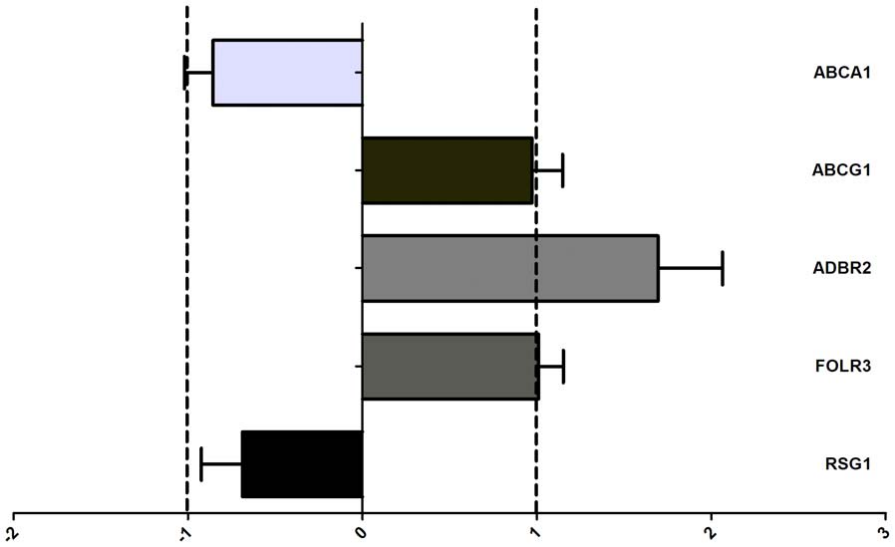
Differential gene expression in patients versus controls determined qPCR in the replication set. The X-axis depicts the relative fold changes in gene expression in the patients compared to controls. depicted as the qPCR 2exp ΔΔCT change. Values are given as mean + SD. A relative fold change of ≤1 or −1 indicates no effect.

Six genes were differentially expressed between the monocytes of patients and controls. The ATP-binding cassette, sub-family A (*ABCA1*; p = 0.0007), ATP-binding cassette, sub-family G (*ABCG1*; p = 0.0001) and Regulator of G-protein signalling 1 (*RGS1*; p = 0.008) were down regulated in the patients. In contrast, the Adrenergic receptor, beta 2 (*ADRB2*; p = 0.007), Folate receptor 3 (gamma) (*FOLR3*; p = 0.04) and Glutathione S-transferase mu 1 (*GSTM1*; p = 0.01) were up regulated in the patients. Differential expression was confirmed by qPCR for *ABCA1* (Hs02565169_s1); *ABCG1* (Hs00245154_m1); *RGS1* (Hs00175260_m1); *ADRB2* (Hs00240532_s1);*FOLR3* (Hs00357145_g1) and *GSTM1* (Hs02341469_m1) by qPCR. No difference in expression of *GSTM1* was found by qPCR. For the other genes, the direction and magnitude of the relative fold changes of the microarray and qPCR experiments were comparable as depicted in figure 1 (p>0.05), except for FOLR3 where the magnitude was significantly higher in the qPCR experiments (p = 0.02). Subsequently, statin and aspirin were started in control subjects and after 6 weeks we ascertained the expression of the 6 genes by qPCR. No significant effect of therapy was observed on the expression of *ABCA1*, *FOLR3* or *RGS1* (Figure 2). However the expression of both *ABCG1* and *ADRB2* decreased significantly following treatment p = 0.001 and p = 0.04 respectively. Finally in the replication group we could confirm the differential expression of *ABCA1*, *ADBR2* and *RSG1* (figure 3).

## Discussion

The aim of our study was to identify novel players in the progression of atherosclerosis. Therefore, we studied gene expression profiles of circulating monocytes of CAD patients and compared the profiles with matched healthy controls and we found that *ABCA1*, *ABCG1*, *RGS1*, *ADRB2* and *FOLR3* were differentially expressed in patients. To match for medication use between patients and controls, statins and aspirin were given to the healthy controls. Differentially expression of two of the five genes was shown to be affected by medication (*ABCG1* and *ADBR2*). Finally we replicate three of the 5 genes found in our replication group.

So far, this is the largest study to determine differential whole gene expression in circulating monocytes in carefully selected early onset myocardial infarction patients and healthy controls. In addition, this is the first study determining the effect of simvastatin and aspirin on selected gene expression *in vivo* [7]–[9].

Previously, Patino and co workers used SAGE to determine gene expression differences in monocytes between two elderly patients with carotid atherosclerosis without a positive family history for CVD and two controls [10]. A total of 297 candidate genes were shown to be 1.5 fold increased in expression and 267 genes had a 1.5 fold decreased expression. To select candidate genes for follow up these researchers applied two additional criteria; low presence in controls and in non-monocytic tissues. This resulted in six candidate genes all of which were regulatory genes or transcriptional factors: Finkel-Biskis-Jinkins osteosarcoma gene (*FOS*), Dual specificity phosphatise 1 (*DUSP1*), nuclear factor of kappa light polypeptide gene enhancer in B cells inhibitor-a (*NFKBIA*) inhibitor of DNA binding 2 (*ID2*), period homolog 1 (*PER1*) and sin3-associated polypeptide (*SAP30*). The authors validated their findings in a second cohort consisting of 25 older patients with carotid atherosclerosis and 19 controls.

Schirmer and co workers compared monocyte gene expression differences between 18 CVD patients and 13 controls [11]. The average age of the event of their partcipants was higher (55.8±7.9 years) and the patients less frequently has a family history of CVD (77.8%). As controls, they chose 13 individuals who had undergone a coronary angiogram because of an intermediate to high risk of CVD but showed no significant coronary abnormalities, which does not exclude CAD [12]. Schirmer et al [8] identified 65 differentially expressed genes. Of this list 6 genes were validated; chemokine (C-C motif) ligand 3 *(CCL3)*, potassium voltage-gated channel, subfamily H (ecg-related), member 2 *(ERG1)*, potassium voltage-gated channel, subfamily H (ecg-related), member 6 *(ERG2)*, FBJ murine osteosarcoma viral oncogene homolog *(FOS)*, zinc finger protein 436 *(ZNF436)* and zinc finger protein 202 *(ZNF202)*. *FOS* was the only one of the 6 genes also reported by Patino et al [10].

The two studies conducted so far on monocyte gene expression *in vivo* have either studied patients with poorly defined phenotypes [10], did not match patients and controls with great care [11], [13] and were smaller in size [10], [11], [14]. Interestingly, except for *FOS*, none of the identified genes were consistently differentially expressed in these two studies. Our *in vivo* finding that statin and aspirin use influences the expression of *ABCG1* and *ADBR2* is novel. For ABCG1 this is in line with an earlier *in vitro* genome wide expression study that showed that treatment of human macrophages derived from peripheral blood for seven days with simvastatin decreased the expression [9].

In total, we report five genes to be differentially expressed in monocytes, one of which might be fully explained by the use of statin and aspirin.

*ABCA1* is located on 21q22.3 and facilitates cellular cholesterol and phospholipid efflux to apolipoprotein A1 in macrophages [15]. Rare loss of function mutations in *ABCA1* are associated with an increased risk of CVD [16]. Decreased expression of this gene, as we found in our MI patients, might result in decreased cholesterol efflux, accumulation of lipids in the vessel wall and thus accelerated atherosclerosis. Decreased expression has also been reported in subjects at increased risk for CVD, because of FH [17]. We did not have patients with FH in our selection. Previous work has shown that statins might down regulate *ABCA1* expression in human macrophages [18]. However, further investigation demonstrated that differential expression was reversed to normal in the presence of cholesterol [9]. This might explain why we did not observe an effect of statin therapy on *ABCA1* expression in the monocytes.

*ABCG1* is located on 9q31.1 and is an ATP binding cassette reporter that participates in the removal of cholesterol from lipid laden macrophages to more premature HDL particles [15]. After statin and aspirin treatment the relative expression of *ABCG1* decreased. This implicates that the observed difference between patients and controls could be due to medication. In line with these findings, statins have shown to induce decreased macrophage expression of *ABCG1* [19].

*RGS1* is located on 1q31 and is a member of the family of regulators of G protein signaling (RGS) proteins and mainly expressed in hematopoietic cells. It may play a major role in the chemokine-mediated homing of lymphocytes to secondary lymphoid organs. Single-nucleotide polymorphisms in this gene have been associated with spondylarteritis, type 1 diabetes mellitus and celiac disease [20]–[22]. In addition they are an independent prognostic marker of disease survival in melanomas [23]. So far, there is no clear association with atherogenesis or CVD.

*ADRB2* is located on 5q31-q32 and is a cell-surface receptor that activates adenylylcyclase by coupling to guanine nucleotide binding proteins (G-proteins).

In vascular smooth muscle cells, the B2 adrenergic receptor mediates vasodilatation in response to adrenergic agonist. In healthy myocardium, the receptor mediates chronotropic and inotropic responses to endogenous and exogenous adrenergic agents [24]. Common polymorphisms in the ADBR2 (rs1042714; Gln27Glu and rs1047213; Arg16Gly) allele are associated with a decreased risk of coronary heart disease incidence in the elderly and agonist-induced down regulation of *ADBR2*. The latter is associated with decreased risk of CVD [24], [25]. In line with this, we observe an increased expression of the *ADBR2* gene in the patients with CVD. After treatment with statin and aspirin the relative expression of *ADRB2* was decreased. This suggests that the increase, which we initially observed, might have been tempered by the medication, and may therefore represent a greater difference between the cases and controls.

*FOLR3* is located on 11q13 and is the secreted form of the folate receptor. It is involved in maintenance of intracellular levels of folate [26]. In pro atherosclerotic hyperlipidemic animal models, increased folate receptor expression is associated with activated macrophages [27]. We found, however, a decreased *FOLR3* expression in our MI patients. However, if the folate receptor is down regulated even in the presence of sufficient circulating folic acid, cellular levels might be low. This could result in increased intracellular homocyteine and signaling pathways resulting in increased atherosclerosis [28].

Our study has several limitations. We hypothesised that in cases enriched for a genetic background for early onset CVD, genetic variants might modulate gene expression in circulating monocytes. However, our expression profiling data, did not reveal shared common variants, since none of the differentially expressed genes are in proximity of any of the newly identified loci for CAD [3]. Nevertheless, the observed differential gene expression could be the result of several other mechanisms, including rare variants and shared environmental factors. These findings are in line with those of Zeller et al. [29] who investigated whether gene sequence variability is linked to phenotypes via gene expression. In a large cohort of 1490 unrelated healthy individuals, they determined genome wide expression in monocytes and attempted to associate this with 10 common risk factors and 675K SNPs. The authors conclude that the transcriptome of circulating monocytes appeared to be of modest help to dissect the relationship between genome variability and complex human traits. With the exceptions of the LPL locus and the 9p21 locus, none of the other cardiovascular risk loci were found to be associated with genome wide expression [29].

The number of genes and relative change in gene expressional profiles between patients and controls were small (<4 fold), which is probably explained by the selection of patients only in a clinically stable phase. Because of the small number of genes differentially expressed; we were not able to identify large differentially regulated pathways. Although our findings in the whole genome expression arrays are strengthened by their validation by qPCR the small fold changes would not make these genes robust biomarkers for CAD.

In our attempt to correct our results for the effects of medication we selected medication which was most frequently used by the cases and based on their mode of action anticipated to modulate monocyte gene transcription. We have not investigated the effect of the less frequently used drugs such as ACE-inhibitors and beta-blockers. Tone et al. studied the effect of angiotensin II type 1 agonist on monocytes *in vivo* [30]. Nineteen genes were differentially expressed after 24 hour incubation of macrophages. However none of these were differentially expressed in our experiments.

In addition, there were two major differences between our study and replication group. The age of onset of disease was much younger in our initial study cohort (36.6 years vs. 43.8). In addition all cases in the study group suffered from a MI whereas only 54% in the replication group.

The differentials expression identified by microarray of *ABCA1*, *RGS1* and *ADBR2* was confirmed by qPCR. The results for *ABCG1* and *FOLR3* were not reproducible by qPCR and therefore require further validation to confirm or refute their differential expression.

In conclusion, we identified three genes in the expression profiles of circulating monocytes which might be associated with early atherogenesis. The fold-changes observed for these genes are relatively small and should be handled with care. However, we note that the findings were replicated with two independent techniques in two independent groups. Further work to confirm these findings is underway in our laboratory.

## Materials and Methods

### Ethics statement

The study was approved by the Institutional Review Board ofthe Academic Medical Centre (AMC) of the University of Amsterdam and conducted according to the declaration of Helsinki.

### Study group

Twenty-two Caucasian males who suffered a myocardial infarction (MI) at a mean age of 36.3±5.7 years with a positive family history for CVD, were selected from our Premature Atherosclerosis (PAS) cohort [31]. Exclusion criteria were a history of Familial Hypercholesterolemia (FH), substance abuse, cancer, diabetes, a current infection or any other co-morbidity. All eligible individuals in this study cohort were approached, starting with the youngest until 22 participants had provided written informed consent. All patients were in a stable condition at least one year after their MI and on statin and aspirin therapy as well as other medication.

Twenty-two, apparently healthy, Caucasian males without a positive family history of CVD were also invited to participate as controls. Controls were matched with the cases for age and current smoking status. Their mean age was 43.1±3.3 years. Because the patients were treated with aspirin and statins, a secondary investigation was carried out in 12 of the healthy controls. They were retested after treatment with simvastatin 40 mg for 6 weeks, and aspirin 100 mg in the last two of the six weeks.

### Replication group

Another twenty-four Caucasian males who suffered from proven CAD at a mean age of 43.8±4.2 years were selected from the PAS cohort. All other in- and exclusion criteria were similar to the study group.

Twenty-four apparently healthy individuals were included matched for age and current smoking status. Their mean age was 49,9±5,1 years.

### Study procedure

Participants visited the AMC simultaneously. After informed consent, medical history was obtained, biometrics were carried out and blood was collected after fasting for at least nine hours. In addition an ECG was performed on the controls to exclude a silent past MI. For the first twelve controls to consent, medication was provided, and they returned after six weeks of medication for collection of further fasting blood samples.

### Monocyte gene expression

Blood was collected for isolation of cells in CTAD tubes (Becton Dickinson, Alphen aan de Rijn, the Netherlands) and centrifuged for 20 minutes at 163 g at 20°C. Subsequently, the buffy coat was collected and monocytes positively selected with CD14+ Dynal beads (Invitrogen, Dynal Biotech, Oslo, Norway) according to manufacturer's instructions.Trizol (Gibco BRL Life Technologies, Breda, the Netherlands) was added and samples were stored in Trizol for a maximum of three months at −80°C. Extracted RNA was quantified with a nanodrop spectrophotometer (NanoDrop Technologies, Delaware, USA) prior to further purification using the RNeasy kit (Qiagen, Valencia, CA, USA). Subsequently, samples were processed using the Illumina RNA Amplification Kit (Ambion Inc., Austin, TX, USA) following the manufacturer's protocol and hybridized to Illumina HumanWG-6 v2 whole-genome expression microarrays (Illumina, Inc., San Diego, CA, USA). In accordance with MIAME (Minimum Information About a Micro-array Experiment) regulations, all data were deposited into ArrayExpress database at www.ebi.ac.uk.

For validation of the study qPCR was used. qPCR was also applied to validate our findings in the replication group. A total of 12 ng of RNA was used for reverse transcription, and amplified using Taqman Transcription Reagents (Applied Biosystems, Foster City, CA, USA). A no template negative control was included and all tests were run in duplicate. For validation therelative expression was compared to the reference geneGlycerinaldehyd-3-phosphat-Dehydrogenase (GAPDH; Hs999999o5_m1). All qPCR probes were ordered from Applied Biosystems by Life Technology, ABI, Amersham, UK. The 96-well plates were read on the Mx4000 Multiplex Quantitative PCR System (Stratagene Inc, La Jolla, California, USA) and data analysed using supplied software. For analysis duplicate wells were treated individually and adaptive baseline and amplification based threshold algorithm enhancements were applied.

### Statistical analysis

Statistical analysis of demographics and laboratory values were performed in SPSS (version 15.0, Chicago, Illinois, USA). Comparisons were performed with an unpaired t-test; percentages between two groups were compared by means of χ2 tests. Variables with a skewed distribution were log-transformed prior to analysis or compared using nonparametric tests. Statistical analysis of the microarray data was performed in R-statistical package and Bioconductor (http://www.r-project.org). Raw data was quantile normalised and filtered to include only features with detection scores above background.

A fold change cut of 1.5 was used. Paired analysis using Student's t-test was carried out across the pairings to identify differentially expressed genes. Statistical analysis of the qPCR data was performed in Graphpad Prism version 5. Relative fold changes due to medication were assessed by performing a Student t-test pre and post medication on the delta delta CTs. Differences between microarray intensities and relative expression as determined by qPCR were also tested by performing a unpaired Student t-test. For all experiments a p-value<0.05 was considered statistically significant.

## Supporting Information

Table S1 **Baseline characteristics Patients and Controls in the Replication study.** Data are presented as mean ± standard deviation (sd) or median (interquartile range)*. Hx indicates history; LDL indicates low-density lipoprotein and HDL indicates high-density lipoprotein. All Lipid values are un-medicated. At time of inclusion all patients received statin therapy.
(DOC)
