# Supplementary material for: Monocyte Gene Expression Signature of Patients with Early Onset Coronary Artery Disease
Source: PLoS One. 2012 Feb 21;7(2):e32166. doi: 10.1371/journal.pone.0032166 (PMC3283726; doi:10.1371/journal.pone.0032166)
Supplement: Table S1 — Baseline characteristics Patients and Controls in the Replication study. Data are presented as mean ± standard deviation (sd) or median (interquartile range)*. Hx indicates history; LDL indicates low-density lipoprotein and HDL indicates high-density lipoprotein. All Lipid values are un-medicated. At time of inclusion all patients received statin therapy. (DOC) [file pone.0032166.s001.doc]

**Supplementary Table 1: Baseline characteristics Patients and Controls in the replication**

**Group**

|  | Patients |  | Controls |
| --- | --- | --- | --- |
|  |  |  |  |
|  | (n=24) |  | (n=24) |
| Baseline characteristics |  |  |  |
| Age ± sd (years) | 50,3 ± 5,3 |  | 49,9 ± 5,1 |
| Age of CAD ± sd (years) | 43,8 ± 4,2 |  | na |
| Family History of early-onset CVD (n,%) | 24 (100) |  | 0 |
| Hx of Hyperlipidemia (n,%) | 3 (13) |  | 0 |
| Hx of Hypertension (n,%) | 5 (21) |  | 0 |
| Hx of Diabetes (n,%) | 0 |  | 0 |
| Hx of Smoking (n,%) | 11 (46) |  | 6 (25) |
| Current Smoking (n,%) | 5 (21) |  | 4 (17) |
|  |  |  |  |
| Biometrics |  |  |  |
| Systolic Bloodpressure ± sd (mmHg) | 138,4 ± 22,0 |  | 129,9 ± 13,7 |
| Diastolic Bloodpressure ± sd (mmHg) | 87,9 ± 13,0 |  | 85,5 ± 11,2 |
| BMI ± sd (kg/m2) | 27,9 ± 3,4 |  | 24,8 ± 2,2 |
|  |  |  |  |
| Total Cholesterol ± sd (mmol/L) | 6,8 ± 1,9 |  | 5,2 ± 0,9 |
| LDL-cholesterol ± sd (mmol/L) | 4,0 ± 1,2 |  | 3,4 ± 0,9 |
| HDL-cholesterol ± sd (mmol/L) | 1,1 ± 0,3 |  | 1,4 ± 0,4 |
| Triglycerides (mmol/L) | 1,6(1,2-2,4) |  | 0,8 (0,6-1,2) |
|  |  |  |  |
| Lipid Lowering Medication (n,%) | 24 (100) |  | 0 |
